# Supplementary material for: Zinc Supplementation Forms Influenced Zinc Absorption and Accumulation in Piglets
Source: Animals (Basel). 2020 Dec 27;11(1):36. doi: 10.3390/ani11010036 (PMC7824504; doi:10.3390/ani11010036)
Supplement: Supplementary file 1 [file animals-11-00036-s001.pdf]

animals

Supplementary Materials: Zinc Supplementation Forms Influenced Zinc Absorption and Accumulation in Piglets

Fen-Fen Liu [1], Md. Abul Kalam Azad [1], Zhi-He Li [1], Jing Li [1,2], Kai-Bin Mo [1,3] and Heng-Jia Ni [1,*]

1 Key Laboratory of Agro-Ecological Processes in Subtropical Region, Hunan Provincial Key Laboratory of Animal Nutritional Physiology and Metabolic Process, Hunan Research Center of Livestock and Poultry Sciences, South Central Experimental Station of Animal Nutrition and Feed Science in the Ministry of Agriculture, Institute of Subtropical Agriculture, Chinese Academy of Sciences, Hunan 410125, China; liufenfen0327@163.com (F.-F.L.); azadmak@isa.ac.cn (M.A.K.A.); 17347124127@189.cn (Z.-H.L.); lijing16099@163.com (J.L.); mokaibin2008@outlook.com (K.-B.M.)
2 Department of Animal Science, Hunan Agriculture University, Hunan 410125, China
3 College of Veterinary Medicine, South China Agricultural University, Guangzhou 510642, China
* Correspondence: nihengjia@isa.ac.cn

Table S1. Daily feed intake and $ZnSO_4$ in drinking water intake.

Day	Zn-Met	$ZnSO_{4,\ feed}$	$ZnSO_{4,\ water}$
1	600 g	600 g	600 g + (90 mg Zn from $ZnSO_4$)
2	600 g	600 g	600 g+ (90 mg Zn from $ZnSO_4$)
3	600 g	600 g	600 g + (90 mg Zn from $ZnSO_4$)
4	600 g	600 g	600 g+ (90 mg Zn from $ZnSO_4$)
5	600 g	600 g	600 g+ (90 mg Zn from $ZnSO_4$)
6	600 g	600 g	600 g+ (90 mg Zn from $ZnSO_4$)
7	700 g	700 g	700 g+(105 mg Zn from $ZnSO_4$)
8	700 g	700 g	700 g+(105 mg Zn from $ZnSO_4$)
9	700 g	700 g	700 g+(105 mg Zn from $ZnSO_4$)
10	800 g	800 g	800 g+(120 mg Zn from $ZnSO_4$)
11	800 g	800 g	800 g+(120 mg Zn from $ZnSO_4$)
12	900 g	900 g	900 g+(135 mg Zn from $ZnSO_4$)
13	900 g	900 g	900 g+(135 mg Zn from $ZnSO_4$)
14	900 g	900 g	900 g+(135 mg Zn from $ZnSO_4$)
15	900 g	900 g	900 g+(135 mg Zn from $ZnSO_4$)
16	1000 g	1000 g	1000 g+ (150 mg Zn from $ZnSO_4$)
17	1000 g	1000 g	1000 g+ (150 mg Zn from $ZnSO_4$)
18	1000 g	1000 g	1000 g+ (150 mg Zn from $ZnSO_4$)
19	1000 g	1000 g	1000 g+ (150 mg Zn from $ZnSO_4$)
20	1000 g	1000 g	1000 g+ (150 mg Zn from $ZnSO_4$)

(90 mg Zn from $ZnSO_4$), a basal diet supplemented with of 90 mg Zn from $ZnSO_4$ dissolved in water.
